# Targeting the COX2/MET/TOPK signaling axis induces apoptosis in gefitinib-resistant NSCLC cells

**DOI:** 10.1038/s41419-019-2020-4

**Published:** 2019-10-14

**Authors:** Juanjuan Xiao, Fei Wang, Hui Lu, Sanpeng Xu, Ling Zou, Qin Tian, Yang Fu, Xuan Lin, Lin Liu, Ping Yuan, Xiaofang Ni, Tengfei Ma, Fanfan Zeng, Peipei Xue, Ruijuan Xiu, Jianmin Zhang, Xinying Ji, Hongbo Hu, Shangyun Lu, Hongtian Dai, Yuan Li, Qian Chu, Xia Zhao, Qiuhong Duan, Feng Zhu

**Affiliations:** 1 Department of Biochemistry and Molecular Biology, School of Basic Medicine, Huazhong University of Science and Technology, Wuhan, Hubei 430030 China; 2 Cancer Research Institute, The Affiliated Hospital of Guilin Medical University, Guilin, Guangxi 541000 China; 3 Department of Pathology, School of Basic Medicine, Huazhong University of Science and Technology, Wuhan, 430030 China; 4 Institute of Pathology, Tongji Hospital, Huazhong University of Science and Technology, Wuhan, 430030 China; 5 Department of General Surgery, The First Affiliated Hospital of Zhengzhou University, Zhengzhou, 450052 China; 6 Internal Medicine, CR&WISCO General Hospital Affiliated to Wuhan University of Science and Technology, Wuhan, 430080 China; 7 Key Lab of Birth Defects and Reproductive Health of National Health and Family Planning Commission, Chongqing Population and Family Planning Science and Technology Research Institute, Chongqing, 400020 China; 8 Henan International Joint Laboratory for Nuclear Protein Regulation, Henan University College of Medicine, Kaifeng, Henan 475004 China; 9 College of Food Science and Nutritional Engineering, China Agricultural University, Beijing, 100083 China; 10 Department of Pathology, National Cancer Center/National Clinical Research Center for Cancer/Cancer Hospital, Chinese Academy of Medical Sciences and Peking Union Medical College, Beijing, 100021 China; 11 Department of Pathology, Union Hospital, Huazhong University of Science and Technology, Wuhan, Hubei 430030 China; 12 Department of Oncology, Tongji Hospital, Huazhong University of Science and Technology, Wuhan, Hubei China

**Keywords:** Kinases, Non-small-cell lung cancer, Growth factor signalling

## Abstract

MET overactivation is one of the crucial reasons for tyrosine kinase inhibitor (TKI) resistance, but the mechanisms are not wholly clear. Here, COX2, TOPK, and MET expression were examined in *EGFR*-activating mutated NSCLC by immunohistochemical (IHC) analysis. The relationship between COX2, TOPK, and MET was explored in vitro and ex vivo. In addition, the inhibition of HCC827GR cell growth by combining COX2 inhibitor (celecoxib), TOPK inhibitor (pantoprazole), and gefitinib was verified ex vivo and in vivo. We found that COX2 and TOPK were highly expressed in *EGFR*-activating mutated NSCLC and the progression-free survival (PFS) of triple-positive (COX2, MET, and TOPK) patients was shorter than that of triple-negative patients. Then, we observed that the COX2-TXA_2_ signaling pathway modulated MET through AP-1, resulting in an inhibition of apoptosis in gefitinib-resistant cells. Moreover, we demonstrated that MET could phosphorylate TOPK at Tyr74 and then prevent apoptosis in gefitinib-resistant cells. In line with these findings, the combination of celecoxib, pantoprazole, and gefitinib could induce apoptosis in gefitinib-resistant cells and inhibit tumor growth ex vivo and in vivo. Our work reveals a novel COX2/MET/TOPK signaling axis that can prevent apoptosis in gefitinib-resistant cells and suggests that a triple combination of FDA-approved drugs would provide a low-cost and practical strategy to overcome gefitinib resistance.

## Introduction

Lung cancer is the most frequent cancer and the most common cause of cancer-related deaths worldwide^1^. Non-small cell lung cancer (NSCLC) represents ~80% ~90% of all lung cancers. Approximately 10–50% of patients with NSCLC harbor *EGFR*-activating mutations such as in-frame deletions in exon 19 (Ex19del, particularly E746-A750del) or missense mutation in exon 21 (L858R)^2^. Although NSCLC with *EGFR*-activating mutations exhibits sensitivity to epidermal growth factor receptor (EGFR) tyrosine kinase inhibitors (EGFR-TKIs), such as gefitinib and erlotinib, most patients treated with EGFR-TKIs develop resistance within 10–14 months^3^.

MET (hepatocyte growth factor receptor, HGFR) overactivation is one of the crucial reasons for TKI resistance. It has been reported that *MET* gene amplification, activating gene mutations, and MET overexpression can lead to MET overactivation, and that these changes were all significantly associated with unfavorable prognosis in NSCLC^4,5^. *MET* encodes a transmembrane tyrosine kinase receptor for hepatocyte growth factor (HGF) and it can also be activated by HGF or by the Src/FAK or EGFR signaling pathway in lung cancer^6,7^. Activated MET interacts with several adaptor proteins, such as STAT3, PI3K, or Src, subsequently activating the mitogen-activated protein kinase and mammalian target of rapamycin pathways to mediate proliferation, apoptosis, and migration^8^. However, the existing molecular regulatory mechanisms and downstream events are not sufficient to explain the pathogenic factors of MET-mediated drug resistance and relapse. More information is needed to explore MET involvement in pathogenesis.

COX2 is overexpressed in inflammatory tissue and many neoplastic tissues^9^. Some studies indicate that its overexpression could result in increased prostaglandin (PG) production and secretion, which in turn activates growth pathways and inhibits apoptotic pathways^10^. COX2 can modulate EGFR transcription through nuclear factor activator protein-1 (AP-1) and promote the malignant transformation of preneoplastic cells^11^. In addition, it was reported that AP-1 could mediate the transcriptional activation of MET^12^. Therefore, whether COX2 could regulate MET transcription through AP-1-inducing TKI resistance drew our attention.

T-lymphokine-activated killer cell-originated protein kinase (TOPK) is associated with histological type, lymph node metastasis, and TNM stage, and is positively correlated with Ki67 and p53 expression in NSCLC^13^. Recently, Li et al.^14^ reported that TOPK silencing inhibited the growth and enhanced the gefitinib sensitivity of lung cancer cells, and TOPK is considered a potential therapeutic target and a prognostic marker for lung cancer. Our previous research found that phosphorylation of TOPK at Y74 and Y272 sites by Src promoted the tumorigenesis of colon cancer^15^. MET is a tyrosine kinase and whether it is upstream of TOPK is unknown. Therefore, the relationship between MET and TOPK in TKI resistance should be studied further.

Here we found that the COX2-TXA_2_ signaling pathway promoted MET transcription through AP-1 and MET phosphorylated TOPK at the Y74 site. Moreover, the activation of this pathway prevented apoptosis and promoted gefitinib resistance in NSCLC. On this basis, the triple combination of COX2 inhibitor celecoxib^16^, TOPK inhibitor pantoprazole^17^, and gefitinib was proven to induce apoptosis in gefitinib-resistant cells and inhibit tumor growth in vitro and in vivo. Our findings may provide a new practical strategy for NSCLC patients with TKI resistance.

## Materials and methods

### Antibodies and reagents

TOPK (sc-293028) and p-AKT (sc-7985) were purchased from Santa Cruz Technology, Inc. (Santa Cruz, CA). β-Actin (ET1701-80) was purchased from Hangzhou HuaAn Biotechnology, Inc. (Hangzhou, ZJ). Antibodies to detect COX2 (#4842), MET (#8198), p-MET (#3077), p-ERK (#9101), ERK (#9102), AKT (#9272), p-c-Jun (#2361), c-Jun (#9165), PARP (#9542), c-PARP (#5625), His (#2365), HA (#3724), and p-Tyrosine (#8954) were purchased from Cell Signaling Technology (Danvers, MA). Horseradish peroxidase-labeled goat anti-mouse IgG (H + L) (E031110) and goat anti-rabbit IgG (H + L) (E031120) were purchased from EarthOx, LLC (San Francisco, CA). Phospho-TOPK at Y74 antibody was prepared by Abgent, Inc. (Suzhou, JS). All antibodies were used following the instructions of the respective manufacturers.

Gentamicin, l-glutamine, epidermal growth factor, HGF, Tris, NaCl, and SDS for molecular biology were purchased from Sigma-Aldrich (St. Louis, MO). U46619, Seratrodast, and Ozagrel HCl were all obtained from ApexBio Technology, LLC (Houston, TX). Celecoxib was purchased from Pfizer (New York, NY). Gefitinib was purchased from Gold Biotechnology (St. Louis, MO). Pantoprazole was obtained from Kaifu Co., Ltd (Wuxi, JS). SR11302 and PGE2 were purchased from Selleckchem, Inc. (Houston, TX).

### Cell culture and transfection

HCC827, MRC-5, H1299, A549, H1650, H1975, and HEK293T cells were purchased from American Type Culture Collection (ATCC; Manassas, VA). The gefitinib-resistant HCC827 (HCC827GR) cell line was kindly provided by Dr. Pasi A. Jänne (Harvard Medical School, Boston, MA) and was authenticated by the Jänne group^18^. Cell lines were authenticated by periodic short tandem repeat profiling and mycoplasma-negative status was confirmed before freezing. All cell lines were thawed and passaged at least three times before initiating experiments. Cells were discarded after 18 passages. Cells were cultured at 37 °C in a 5% CO_2_ humidified incubator following the ATCC protocols. MRC-5 human normal lung fibroblasts were grown in Eagle’s minimum essential medium (Gibco, Grand Island, NY) with 10% fetal bovine serum (FBS; Gibco, Grand Island, NY) and A549 human lung cancer cells were cultured with F-12K medium (Sigma, St. Louis, MO) containing 10% FBS. All other human lung cancer cells were grown in RPMI-1640 medium (Gibco, Grand Island, NY) supplemented with 10% FBS. HEK293T cells (stably expressing the SV40 large T antigen in HEK293 cells) were cultured in Dulbecco’s modified Eagles’ medium (Gibco, Grand Island, NY) supplemented with 10% FBS.

When cells reached 60% confluence, transfection was performed using Simple-fect (Signaling Dawn Biotech, Wuhan, HB) following the manufacturer’s instructions. For stable transfection experiments, G418 (Sigma, St. Louis, MO) or hygromycin (Thermo Fisher, Waltham, MA) was added for stable clone selection. After 3 weeks, the individual clones were ring-isolated. Expression of the protein was verified by western blotting analysis.

### Clinical data and IHC analysis of a tissue array

One hundred fifty-three cases of NSCLC with activating mutations in the *EGFR* gene, including a deletion in exon 19 and an L858R mutation in exon 21, were collected from archival files of Wuhan Tongji Hospital dating from 2013 to 2016. NSCLC specimens and matched adjacent normal tissues were used to construct a tissue microarray (TMA). Ethical approval was obtained from the medical ethics committee of Tongji Medical College, Huazhong University of Science and Technology. Paraffin-embedded tissues were sectioned at 5 µm thickness. Slides were baked at 60 °C for 1 h and were deparaffinized, rehydrated, and treated with 3% hydrogen peroxide for 10 min. Antigen retrieval was performed in citrate buffer pH 6.0 in a steamer for 2 min or in Tris-EDTA buffer pH 9.0 at 100 °C for 20 min. After the slides were blocked with 5% bovine serum albumin in phosphate-buffered saline (PBS) for 30 min, tissue sections were incubated overnight at 4 °C with the indicated primary antibodies. The PBS and mouse or rabbit IgG1 (Santa Cruz Biotechnology, CA) were used as blank and negative controls. The sections were photographed using an Olympus Imaging System Microscope (BX51, Olympus, Tokyo). Immunohistochemical (IHC) staining was evaluated simultaneously by two pathologists who did not know the clinicopathological features of the patients.

### Western blotting analysis and immunoprecipitation

Cells were collected and lysed in RIPA buffer (1 × PBS, 1% Nonidet P-40, 0.5% sodium deoxycholate, 0.1% SDS, 1 mmol/L Na_3_VO_4_, and 1 mmol/L aprotinin and 1 mmol/L phenylmethylsulfonyl fluoride). Then, the samples were sonicated 15 s three times and centrifuged at 12,000 r.p.m. for 10 min. The quantity of protein was determined by the Bradford method. After that, the samples were separated on a 7%–15% SDS-polyacrylamide gel electrophoresis (PAGE) gel, transferred onto a polyvinylidene difluoride membrane (Millipore, Billerica, MA) and subsequently visualized by chemiluminescence (BIO-RAD, Hercules, CA) in triplicate. The samples for immunoprecipitation were collected in 1% CHAPS (3-[(3-Cholamidopropyl)dimethylammonio]propanesulfonate) instead of RIPA buffer. Equal amounts of protein (1–2 mg) were subjected to immunoprecipitation following the manufacturer’s suggested protocol.

### Anchorage-independent cell transformation assay

Different cell lines (8 × 10^3^/well) in a six-well plate were exposed or not exposed to different drugs and cultured in 1 ml of 0.33% BME (Eagle basal medium, Sigma, St. Louis, MO) agar (Sigma, St. Louis, MO) containing 10% FBS over 3 ml of 0.5% BME agar containing 10% FBS. The cells were maintained in a 37 °C, 5% CO_2_ incubator for 5–10 days and then their colonies were counted and scored using Image-Pro Plus software.

### In vitro growth inhibition assay

HCC827GR cells were seeded in each well of a 96-well plate and were cultured in RPMI-1640 medium with 10% FBS for 24 h. Then, different drugs at the indicated doses were added to the medium for an additional 24, 48, or 72 h. The inhibitory effects of different groups on cell growth were examined using the MTT (3-(4,5-dimethylthiazol-2-yl)-2,5-diphenyltetrazolium bromide) (Sigma, St. Louis, MO) viability assay, according to the manufacturer’s instructions. All the experiments were performed in triplicate and the mean absorbance values were calculated.

### Prostaglandin determination

The measurement of PGs (PGD_2_, PGE_2_, PGF_2_, PGI_2_, and TXA_2_) in the cell culture medium was conducted using enzyme immunoassay kits from Cayman Chemical Company (Ann Arbor, MI). In brief, cells were plated in six-well plates. When cells reached 80% confluence, 1 ml fresh medium with or without reagents was added and cells were further incubated for a different time. Then, the supernatants were collected for PG measurement following the manufacturer’s instructions.

### Flow cytometry analysis

Cell apoptosis was determined using the Annexin V–fluorescein isothiocyanate (FITC)/Propidium Iodide (PI) Apoptosis Detection Kit (Elabscience, Wuhan, HB) following the manufacturer’s suggested protocol. Cells (2 × 10^5^/well) were seeded in a six-well plate and cultured at 37 °C and 5% CO_2_ for 12 h. After treatment with gefitinib for 24 h, cells were collected and washed with PBS. Then, the cells were incubated for 15 min at room temperature with Annexin V–FITC plus PI according to the protocol. Apoptosis was analyzed by a FACSCalibur flow cytometer (BD Biosciences, San Jose, CA, USA).

### In vitro kinase assay

The MET active kinase, ATP, and 10 × kinase buffer were purchased from Millipore Corporation (Billerica, MA). His-TOPK-WT and His-TOPK-Y74F were expressed in *Escherichia coli* BL21 bacteria as before^15^. Peptides were synthesized by GL Biochem Ltd (Shanghai, SH). The inactive substrate (2 μg) and the active kinase (0.2 μg in a 30 μl reaction) were incubated at 32 °C for 1 h in 1 × kinase buffer containing 100 μmol/L ATP or 1 μCi [γ-^32^P]-ATP (China Isotope & Radiation Corporation, Beijing, BJ). The samples were added to 5 × SDS buffer, and then resolved by SDS-PAGE and visualized by autoradiography or western blot.

### Combined drug analysis

The combined effect was evaluated by MTT assay at the nonconstant ratio of each drug. CompuSyn (ComboSyn, Inc., Paramus, NJ) was used to assess the interaction of the drug combinations for synergy/additivity/antagonism using the Combination index (CI) value from the Chou–Talalay method^19^. CI values of <1, 1, and >1 indicate synergistic, additive, and antagonistic effects, respectively.

### In vivo study

Male NOD-Prkdc^scid^ Il2rg^null^ (NPSG) mice (18–20 g, 6 weeks of age, Weishanglituo Company, Beijing) were randomly divided into three groups (*n* = 10 per group). HCC827GR cells (5 × 10^6^/0.1 ml RPMI-1640 medium, 0.1 ml Matrigel) were inoculated subcutaneously into the right flank of each mouse. When tumors reached an average volume of 100 mm^3^, vehicle control, gefitinib (20 mg/kg), or gefitinib (20 mg/kg) plus celecoxib (30 mg/kg) plus pantoprazole (150 mg/kg) was administered by intraperitoneal injection three times a week for 3 weeks. Double blinding was done in this experiment. Tumor volume was calculated using the ellipsoid formula (length × width × height × 0.52). Animal maintenance and experimental procedures were approved by the Animal Care Committee of Wuhan Servicebio Technology Co., Ltd (Wuhan, China).

### Statistical analysis

All quantitative data except for the combined drug analysis are expressed as the mean values ± SD of at least three independent experiments or samples. Significant differences were determined by Student’s *t-*test or one-way analysis of variance. The Pearson’s correlation was used to measure the strength of association between two variables. All statistical tests were two-sided and *P* *<* 0.05 was considered significant (*P < 0.05, **P < 0.01, ***P < 0.001).

## Results

### COX2 is the upstream regulator of MET in HCC827GR cells and *EGFR*-activating mutated NSCLC

**Fig. 1:**
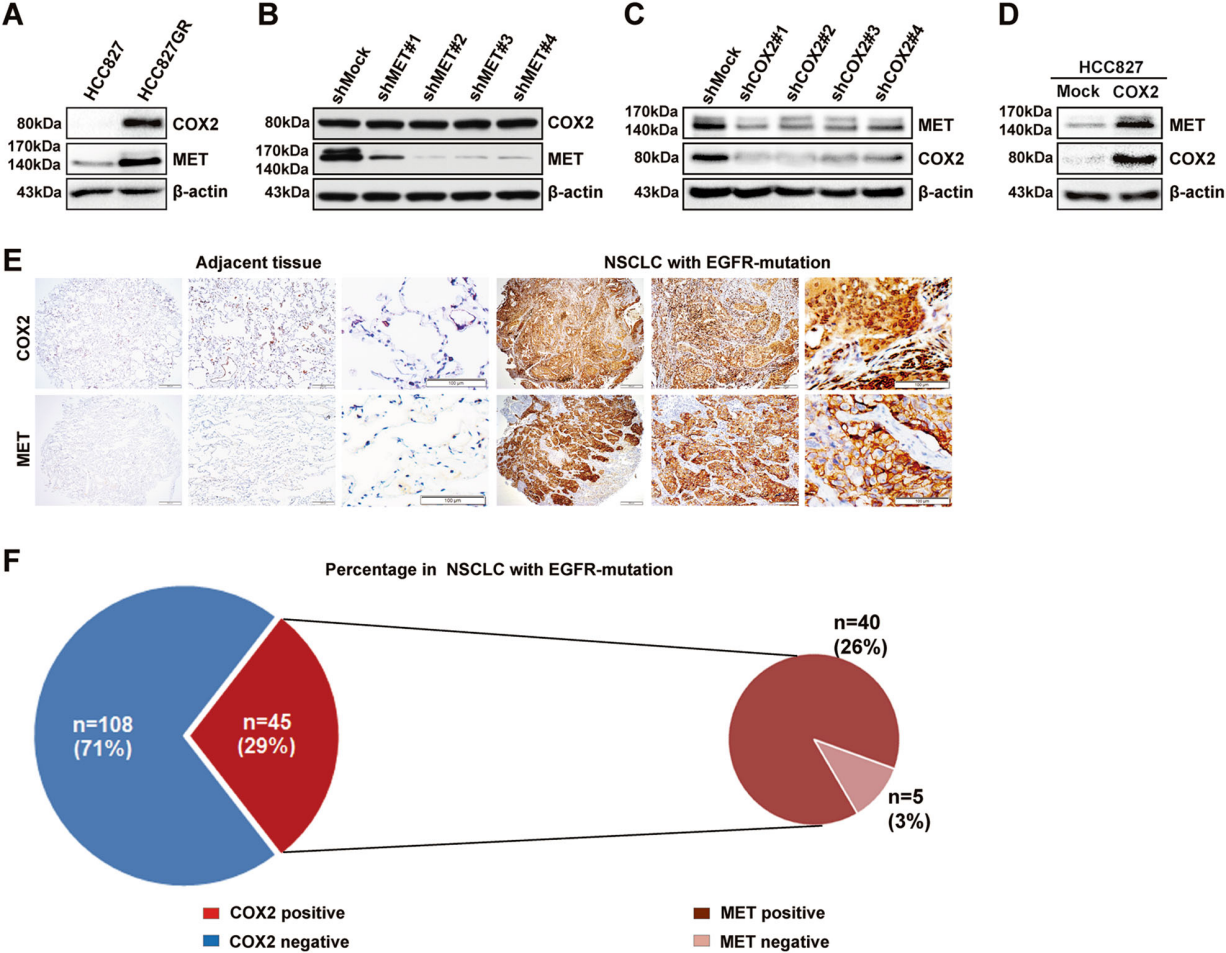
COX2 regulates MET in HCC827GR cells and *EGFR*-mutated NSCLC. The levels of COX2 and MET were shown in HCC827GR cells **a**, HCC827GR cells with *MET/COX2* gene silencing **b**, **c**, and HCC827 cells stably expressing COX2 **d** by western blotting analysis. **e**, **f** IHC analysis of COX2 and MET expression in TMA. Data are representative of results from triplicate experiments. Magnifications, ×40, ×100, ×200

To confirm whether COX2 could regulate MET expression in NSCLC, we first analyzed the expression of COX2 in HCC827 and HCC827GR cells (*MET* amplification, gefitinib resistance^18^). The results in Fig. 1a showed that COX2 expression was significantly higher in HCC827GR cells than in HCC827 cells. After *MET* was knocked down in HCC827GR cells, the expression of COX2 did not change (Fig. 1b). However, after silencing *COX2* in HCC827GR cells, the expression of MET was dramatically decreased (Fig. 1c). In turn, the expression of MET was increased in HCC827 cells stably overexpressing COX2 (Fig. 1d). These results suggested that COX2 acted as an upstream regulator of MET in HCC827GR cells, but MET could not regulate COX2.

Next, we further examined the expression of COX2 and MET in the TMA as described previously by IHC staining. The results showed that the protein levels of COX2 or MET were increased in *EGFR*-activating mutated NSCLC compared with adjacent tissues (Fig. 1e). The positive expression rate of COX2 was 29% in these NSCLC patients and it was especially noteworthy that the positive expression rate of MET was as high as 89% in the COX2-positive NSCLC patients (Fig. 1f). This finding revealed that COX2 could also be an upstream regulator of MET in NSCLC patients with both COX2 and MET-high expression.

### TXA_2_ levels were significantly elevated in HCC827GR cells and the COX2-TXA_2_ signaling pathway modulates MET transcription through AP-1

**Fig. 2:**
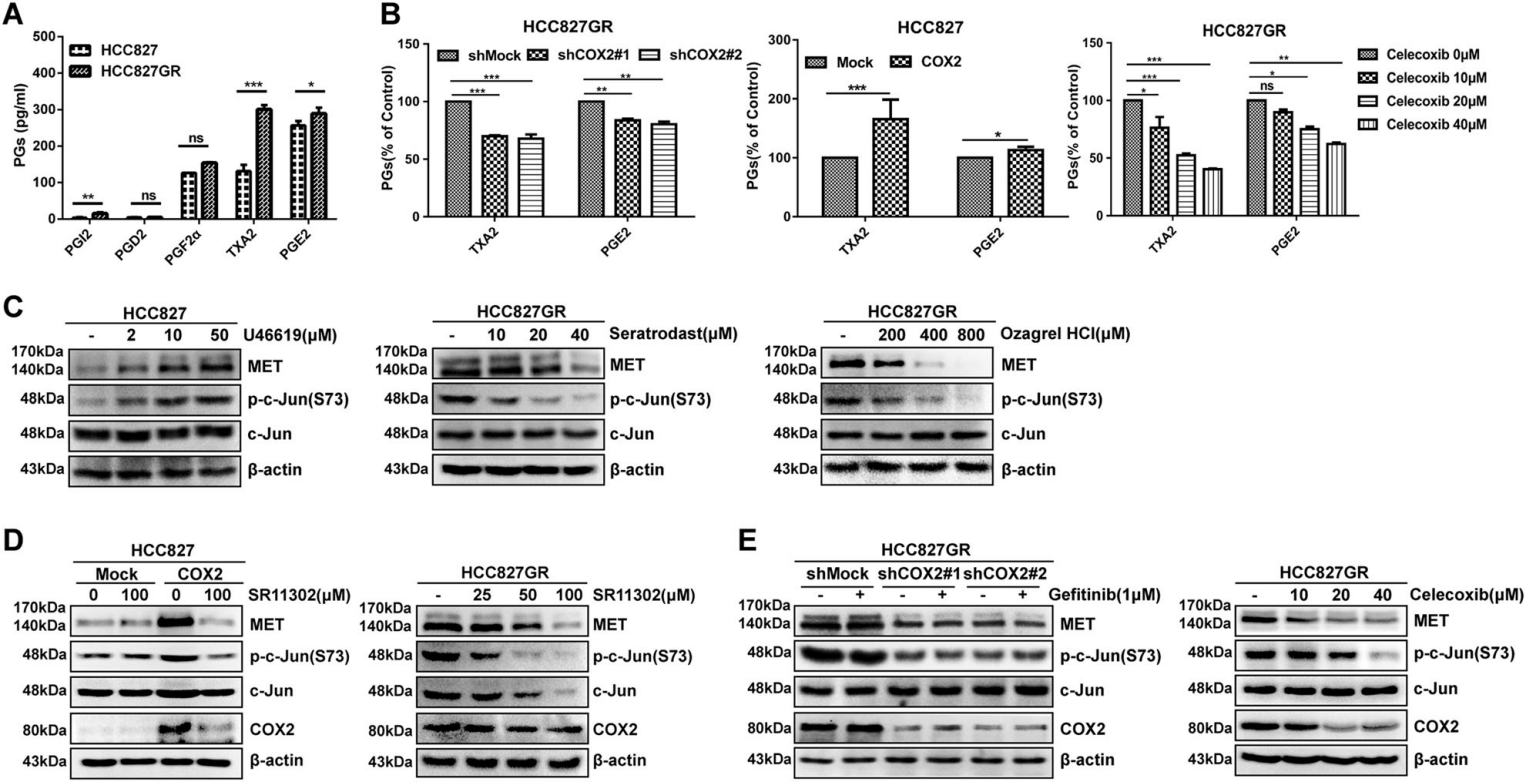
TXA_2_ levels are elevated in HCC827GR cells and the COX2-TXA_2_ signaling pathway modulates MET transcription through AP-1. **a** The levels of PGs were measured in the supernatants from HCC827 and HCC827GR cells. **b** Measurement of TXA_2_ and PGE_2_ in supernatants from shCOX2 HCC827GR cells (left), HCC827 cells stably expressing COX2 (middle), or HCC827GR cells treated with celecoxib for 48 h at different doses (right). **c** HCC827 cells were treated with U46619 for 48 h at different doses (left); HCC827GR cells were treated with different doses of Seratrodast (middle) or Ozarel HCl (right) for 48 h. **d** HCC827 cells stably expressing COX2 (left) or HCC827GR cells (right) were treated with SR11302 for 48 h. **e** The stable shCOX2 HCC827GR cells were treated with gefitinib (1 μΜ) for 6 h (left); HCC827GR cells were treated with different doses of celecoxib for 48 h (right). Those samples were resolved by SDS-PAGE and visualized by western blotting. Data are representatives of results from triplicate experiments. *P < 0.05, **P < 0.01, ***P < 0.001

COX2, as a rate-limiting enzyme, can convert arachidonic acid to PGs^20^. To further explore which PGs participated in the regulation of MET, five major bioactive PGs of the supernatant fractions of HCC827 and HCC827GR cells were measured by enzyme-linked immunosorbent assay. The results showed that TXA_2_ and PGE_2_ were the abundant PGs in HCC827GR cells. Compared with those in HCC827 cells, the levels of TXA_2_, PGE_2_, and PGI_2_ were significantly elevated in HCC827GR cells, whereas PGF_2α_ and PGD_2_ levels did not change significantly. Intriguingly, the TXA_2_ level changed the most, with a striking threefold enhancement (Fig. 2a). Furthermore, the levels of TXA_2_ and PGE_2_ (Fig. 2b left) from the supernatant fractions of HCC827GR cells with shCOX2 were decreased and COX2 overexpression in HCC827 cells increased the levels of TXA_2_ and PGE_2_ (Fig. 2b, middle). After HCC827GR cells were treated with celecoxib, the levels of TXA_2_ and PGE_2_ gradually decreased in a dose-dependent manner (Fig. 2b, right). Based on the above results, the high levels of TXA_2_ and PGE2, especially TXA_2_, may play essential roles in HCC827GR cells. To confirm the role of TXA_2_ in gefitinib resistance, three experiments were designed. First, HCC827 cells were treated with different concentrations of TXA_2_ receptor agonist (U46619) for 48 h and the level of MET was detected by western blotting. The results showed that the expression of MET gradually increased in a dose-dependent manner after U46619 treatment (Fig. 2c, left). Second, the expression of MET was gradually decreased in a dose-dependent manner in HCC827GR cells treated with TXA_2_ receptor antagonist (Seratrodast) or the inhibitor of TXA_2_ synthase (Ozagrel HCl) for 48 h (Fig. 2c, middle or right). These results suggest that among five PGs, TXA_2_ is the critical mediator that regulates MET expression in HCC827GR cells.

Previously, it was reported that AP-1 was one of the potential transcription factors for MET^12^. Moreover, the TXA_2_ receptor could lead to the activation of AP-1 by activating a protein kinase C-dependent pathway^21^. We hypothesized that the COX2-TXA_2_ signaling pathway might modulate MET transcription through AP-1. To verify whether the COX2-TXA_2_ signaling pathway increases MET expression through AP-1 in HCC827GR cells, we tested the AP-1 activity of HCC827GR cells using a phospho-c-jun (S73) antibody by western blotting. The results showed that the level of p-c-jun (S73) was enhanced in a dose-dependent way with U46619 treatment and attenuated with Seratrodast or Ozagrel HCl treatment (Fig. 2c). Meanwhile, the expression of MET was also strikingly decreased in HCC827GR cells or HCC827 cells stably overexpressing COX2 with AP-1 inhibitor (SR11302) treatment (Fig. 2d). Lower AP-1 activity and MET levels were observed in COX2 knockdown or celecoxib-treated HCC827GR cells (Fig. 2e). Hence, these results verified that the COX2-TXA_2_ signaling pathway increased MET expression through AP-1.

### The expressions of TOPK and MET are positively correlated in HCC827GR cells and *EGFR*-activating mutated NSCLC

**Fig. 3:**
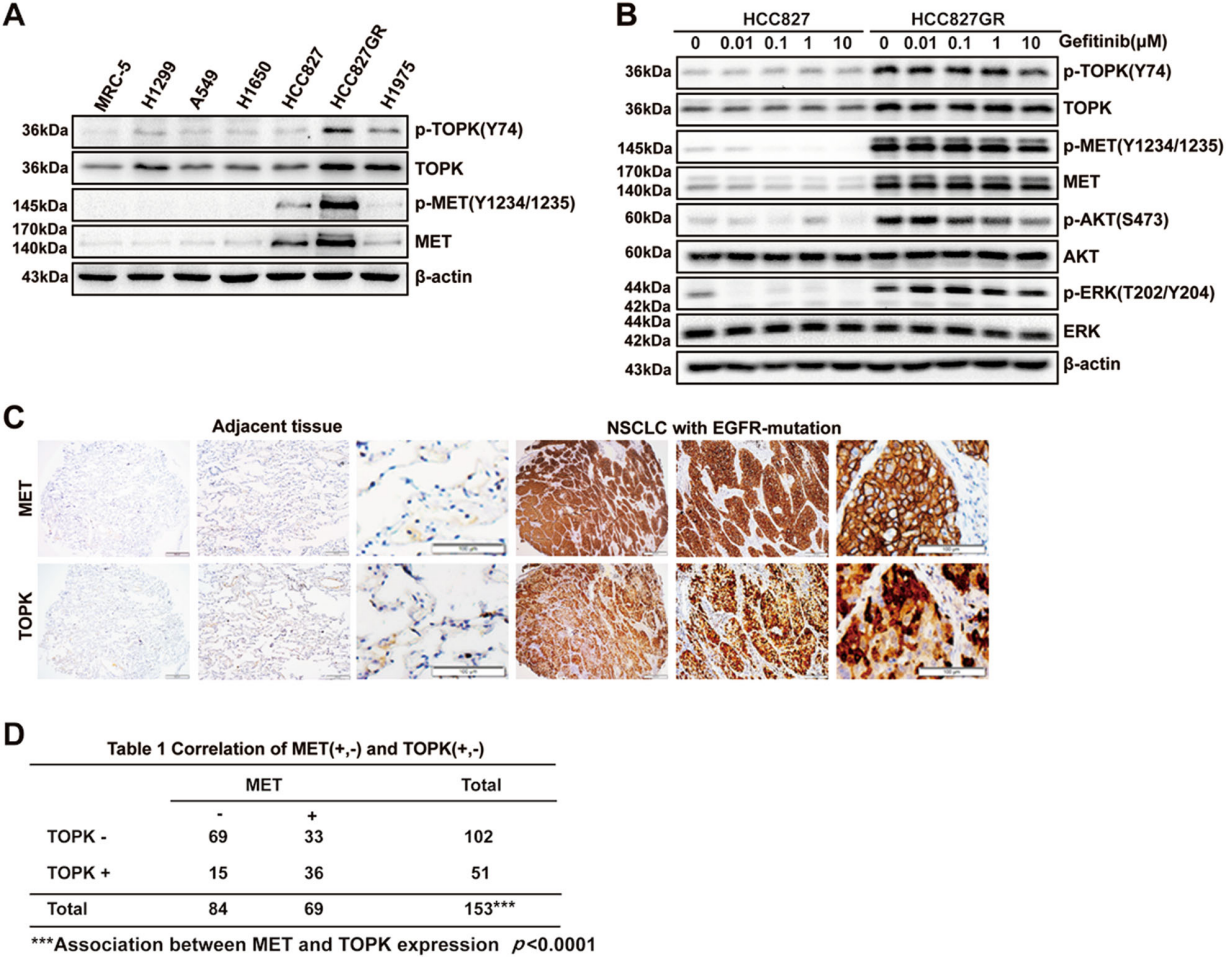
The expression levels of TOPK and MET are positively correlated in *EGFR*-mutated NSCLC. **a** Western blotting analysis in different lung cancer cells. **b** HCC827 and HCC827GR cells were exposed to increasing concentrations of gefitinib for 6 h. The samples were separated by SDS-PAGE and visualized by western blotting. **c** IHC analysis of MET and TOPK expression in TMA. Magnifications, ×40, ×100, ×200. **d** The correlation between MET and TOPK expression in *EGFR*-activating mutated NSCLC was analyzed. Data are representative of results from triplicate experiments. ***P *<* 0.001; *R* = 0.1801

TOPK was involved in the growth of lung cancer cells and could be activated by tyrosine kinase Src^14,15^. Therefore, the role of TOPK in gefitinib-resistant NSCLC with MET overactivation attracted our attention. Our results showed that TOPK and p-TOPK (Y74) levels were much higher in HCC827GR cells than in other lung cancer cell lines and a normal lung cell line (Fig. 3a). Unlike that in the parental HCC827 cells, the phosphorylation of TOPK, MET, and ERK1/2 in the HCC827GR cells was maintained in the presence of gefitinib (Fig. 3b). In addition, IHC analysis showed that the protein level of TOPK was increased in *EGFR*-activating mutated NSCLC compared with that in adjacent tissues (Fig. 3c). TOPK staining was positive in 121 (79%) of 153 samples. Importantly, MET and TOPK were highly and positively correlated (*p* < 0.0001; *R* = 0.1801) in *EGFR-*activating mutated NSCLC patients (Fig. 3d). These results demonstrated that the increased activity of TOPK is related to gefitinib resistance, and that the expression of TOPK has a positive correlation with that of MET in EGFR-activating mutated NSCLC.

### MET phosphorylates TOPK at the Y74 site in vitro and ex vivo

**Fig. 4:**
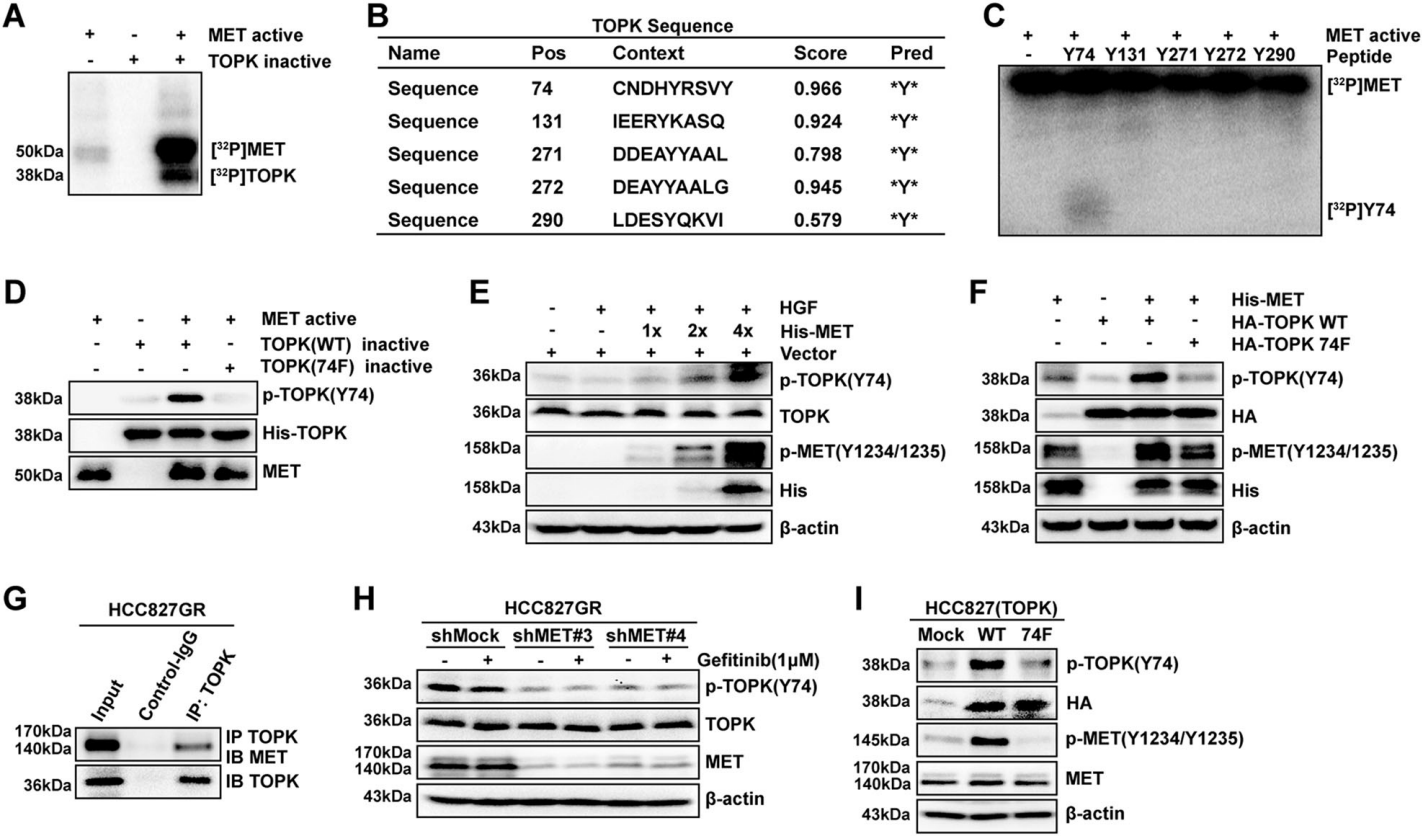
MET phosphorylates TOPK at the Y74 site in vitro and ex vivo. **a** Active MET phosphorylated inactive TOPK-WT in vitro in the presence of [γ-^32^P] ATP as visualized by autoradiography. **b** Potential phosphorylated Tyr sites of TOPK were predicted by the NetPhos 3.0 software program. **c** Synthesized peptides containing potential Tyr **b** sites were used as substrates in an in vitro kinase assay with active MET in the presence of [γ-^32^P] ATP and the results were visualized by autoradiography. **d** Active MET phosphorylated inactive TOPK-WT or 74F in vitro in the presence of ATP. Then, the samples were analyzed by western blotting. **e** MET promoted the phosphorylation of TOPK in HEK293T cells induced by HGF after transfection with His-MET in a dose-dependent manner (HGF 40 ng/ml; 15 min). **f** His-MET was cotransfected in HEK293T cells with wild-type TOPK (HA-TOPK WT) or Y74-mutated TOPK (HA-TOPK 74F). **g** TOPK bound with endogenous MET of HCC827GR cells by IP assay. **h** The stable shMock and shMET in HCC827GR cells were treated with gefitinib (1 μΜ) for 6 h. **i** HCC827 cells stably overexpressing Mock, HA-TOPK WT, and HA-TOPK 74F were cultured and analyzed by western blotting. The above samples were collected and analyzed by western blotting. Data are representative of results from triplicate experiments

Next, we wondered whether MET, as a tyrosine kinase, could phosphorylate TOPK directly. The results of the in vitro kinase assay indicated that MET could phosphorylate TOPK in vitro (Fig. 4a). The potential tyrosine phosphorylation sites of TOPK were predicted by NetPhos 3.0 (Fig. 4b). Five high-scoring peptides were synthesized commercially (Y74, Y131, Y271, Y272, and Y290) and individually incubated with active MET in the presence of [γ-^32^P] ATP in an in vitro kinase assay. The data showed that the Y74 peptide of TOPK was notably phosphorylated by MET (Fig. 4c). Subsequently, the wild-type TOPK (WT) and Y74F TOPK (74F) proteins were purified from *E. coli* and were used as substrates for active MET in an in vitro kinase assay. Western blotting analysis using the prepared specific p-TOPK (Y74) antibody confirmed that MET could phosphorylate TOPK at the Y74 site (Fig. 4d).

We further verified that MET could phosphorylate TOPK in cells. First, MET was overexpressed in HEK293T cells and then the cells were stimulated with HGF. The results showed that the phosphorylation level of endogenous TOPK at Y74 was gradually increased in a dose-dependent manner with increasing amounts of transfected MET plasmid (Fig. 4e). Then, TOPK WT plasmid (HA-TOPK WT) or HA-TOPK 74F plasmid was cotransfected into HEK293T cells with MET plasmid (His-MET). The results showed that the level of p-TOPK (Y74) increased in the group cotransfected with HA-TOPK WT (Fig. 4f). Subsequently, endogenous TOPK was immunoprecipitated from HCC827GR cells and then MET was detected by western blotting. The results indicated that TOPK could coimmunoprecipitate with MET in HCC827GR cells (Fig. 4g). After that, the level of endogenous p-TOPK (Y74) was decreased in *MET* knockdown HCC827GR cells (Fig. 4h). Moreover, it was also revealed that the level of p-TOPK (Y74) was increased in TOPK-WT-overexpressing HCC827 cells and the level of p-TOPK (Y74) was obviously decreased in TOPK-74F cells (Fig. 4i). These results thus far indicated that the phosphorylation of TOPK by MET at the Y74 site existed in cells.

### The COX2/MET/TOPK signaling axis inhibits cell apoptosis and promotes the anchorage-independent growth ability of HCC827 cells

**Fig. 5:**
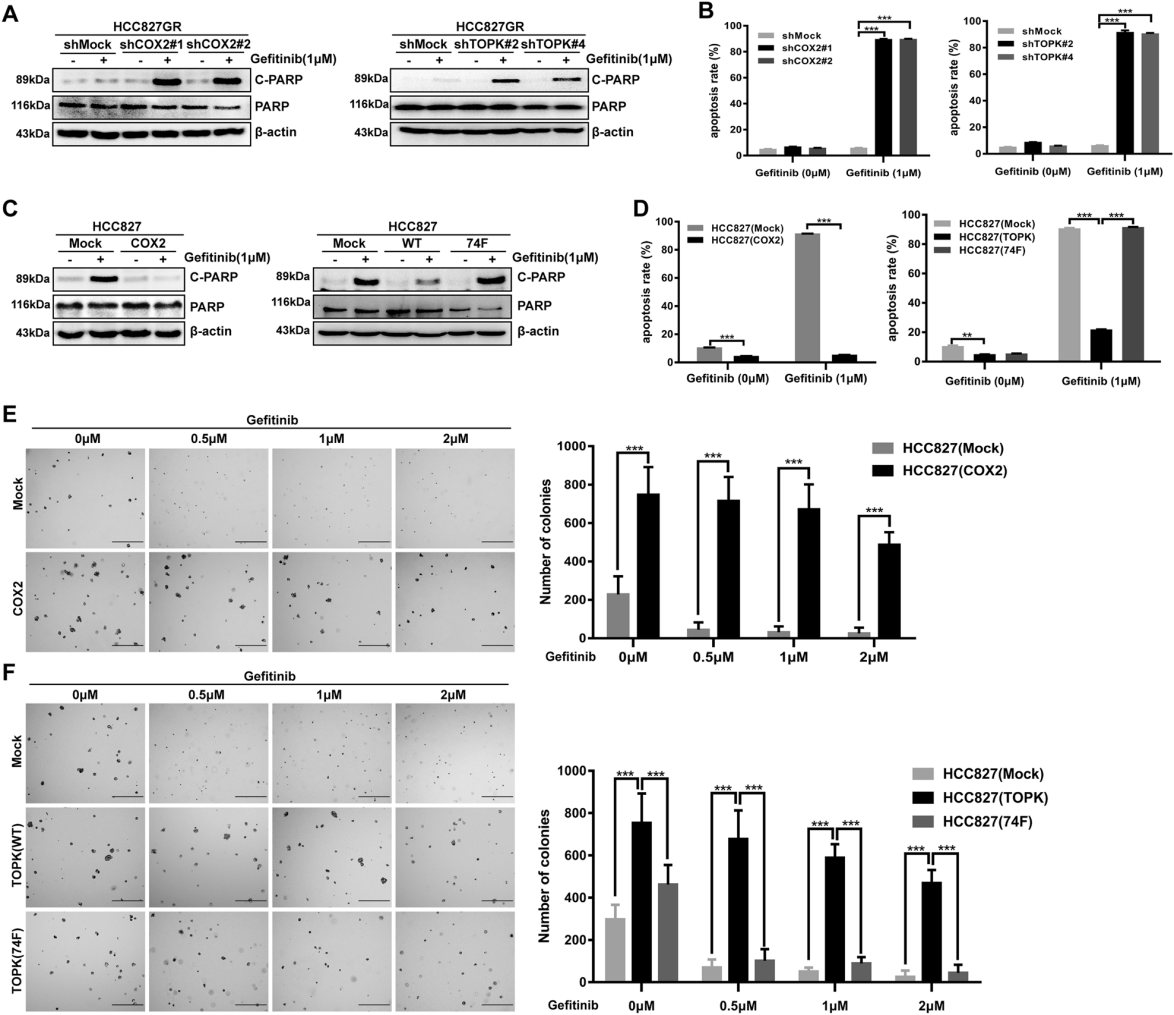
The COX2/MET/TOPK signaling axis inhibits cell apoptosis and promotes the anchorage-independent growth ability of HCC827 cells. **a**, **b** *COX2* (left) or *TOPK* (right) gene silencing in HCC827GR cells promoted cell apoptosis with gefitinib (1 μΜ) treatment for 24 h. Total and cleaved PARP were detected by western blotting analysis **a**. Apoptotic cells were detected by Annexin V–FITC and PI double staining and analyzed by flow cytometry **b**. **c**, **d** Stably expressing COX2 (left) or HA-TOPK WT (right) in HCC827 cells inhibited cell apoptosis; stably expressing HA-TOPK 74F (right) promoted cell apoptosis. The cells were treated with gefitinib (1 μΜ) for 24 h. Total and cleaved PARP and apoptotic cells were detected as same as **a**, **b**. **e**, **f** Transfectants of HCC827 (Mock), (COX2), (TOPK-WT) or (74F) were compared for colony formation by soft agar assay with gefitinib treatment (0 μΜ, 0.5 μΜ, 1 μΜ, 2 μΜ). Scale bar: 500 μm. **P < 0.01, ***P < 0.001. Data are representative of results from triplicate experiments

From the above results, we found that COX2 regulated MET by AP-1 and MET phosphorylated TOPK at the Y74 site. Then, we further explored the effect of the COX2/MET/TOPK signaling pathway on gefitinib resistance. The cleavage of PARP (a marker of apoptosis) was increased in knockdown COX2 or TOPK HCC827GR cells after 1 µM gefitinib treatment for 24 h (Fig. 5a). Flow cytometry revealed that knockdown of COX2 or TOPK promoted apoptosis of HCC827GR cells (Fig. 5b, and Fig. S1A and S1B). Meanwhile, decreased PARP cleavage was seen in HCC827 cells stably overexpressing COX2 or TOPK after treatment with gefitinib (1 µM, 24 h) (Fig. 5c). The expression of cleaved PARP was recovered in the group stably overexpressing TOPK-74F (Fig. 5c, right). In addition, flow cytometry showed that overexpression of COX2 or TOPK in HCC827 cells inhibited cell apoptosis and the apoptosis rate of HCC827 cells stably overexpressing TOPK-74F was increased compared with HCC827 cells stably overexpressing TOPK after treatment with gefitinib (1 µM, 24 h) (Fig. 5d, and Fig. S1C and S1D). Based on these data, the anchorage-independent colony-formation ability of stable cell lines was then tested. The results showed that gefitinib strongly inhibited HCC827 (Mock) cell growth as expected at different concentrations (0.5 µM, 1 µM, or 2 µM). Compared with the control group, the colonies formed by HCC827 (COX2) cells were significantly greater in number and larger in size, and could not be entirely inhibited by gefitinib (Fig. 5e). Similarly, the colonies formed by HCC827 (TOPK-WT) cells were also more numerous and larger in size than those formed by HCC827 (Mock) cells with or without gefitinib treatment. Moreover, fewer and smaller colonies were formed by the HCC827 (TOPK-74F) cells (Fig. 5f). These results indicated that COX2 and TOPK could inhibit the apoptosis of HCC827 cells and promote the anchorage-independent growth ability of HCC827 cells. This suggests that the inhibition of COX2 and TOPK may overcome resistance to gefitinib.

### The triple combination of drugs strongly suppresses cell growth and colony formation accompanied by induction of apoptosis in vitro and in vivo

**Fig. 6:**
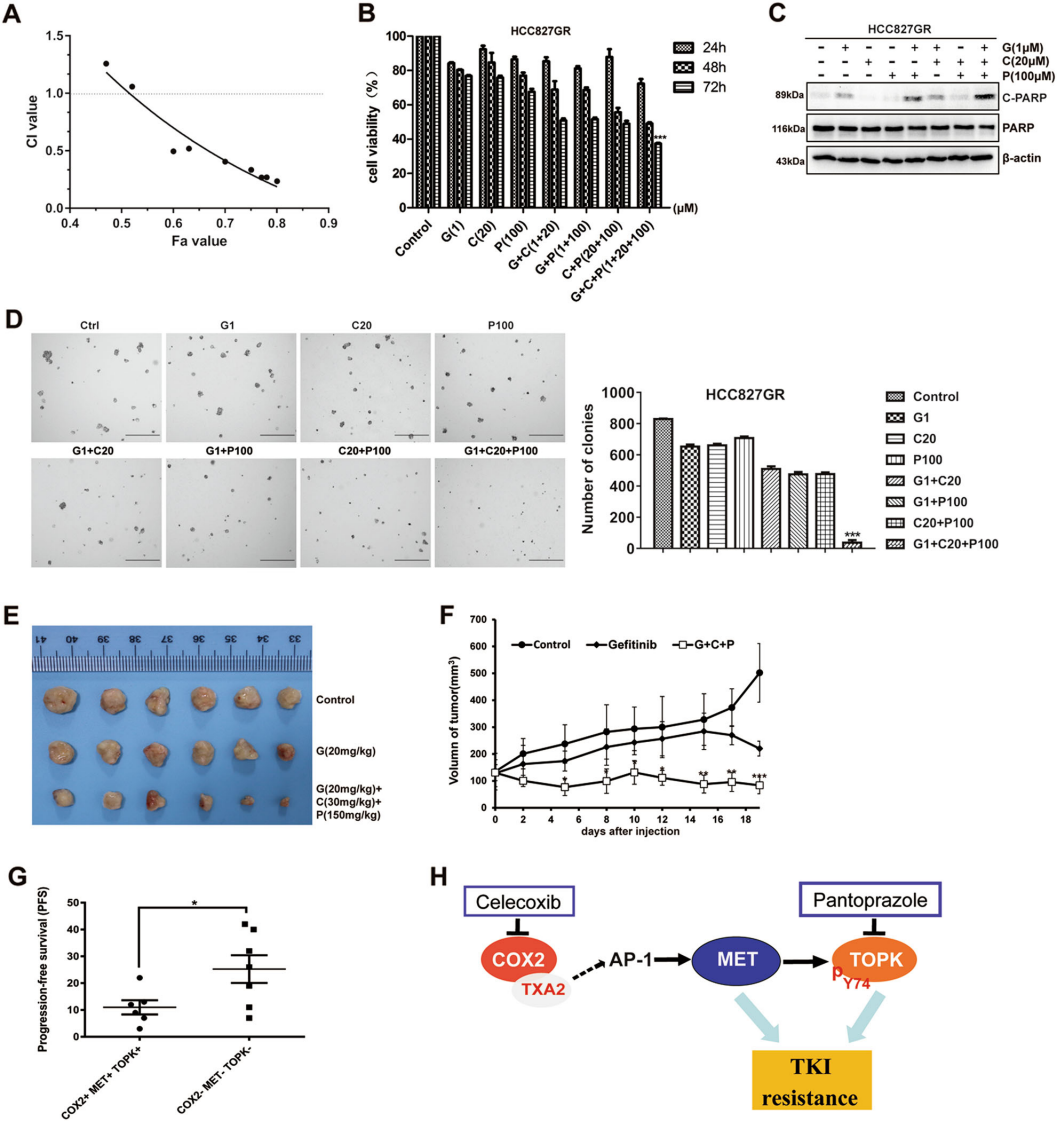
Effects of combined treatment with gefitinib, celecoxib, and pantoprazole in vitro and in vivo. **a** Combined effects were measured using CI values. Quantification of the potency of the different triple combinations using the Chou–Talalay method as described in the Materials and Methods section. Fa: Fraction affected. CI values < 1.0 correspond to synergistic inhibition effects, and the CI of gefitinib (1 μΜ), celecoxib (20 μΜ), and pantoprazole (100 μΜ) in HCC827GR was 0.495. **b** HCC827GR cells were treated with gefitinib (1 μΜ), celecoxib (20 μΜ), and pantoprazole (100 μΜ) alone, with the three distinct combinations of two inhibitors and with the triple combination for 24, 48, or 72 h. Then, the effects on cell growth were measured by MTT assay. Bars represent the SD of experiments repeated in triplicate. ***P < 0.001 for the triple combination of drugs vs. either control or drug alone or other two drugs. **c** HCC827GR cells were treated with the same drugs as **b** for 24 h and the samples were analyzed by western blotting. **d** The colonies in soft agar were compared in HCC827GR cells treated with gefitinib, celecoxib, or pantoprazole. Scale bar: 500 μm. ***P *<* 0.001 for the triple combination of drugs vs. either control or drug alone or other two drugs. **e**, **f** Mice were treated by intraperitoneal injection with vehicle, gefitinib, or gefitinib plus celecoxib plus pantoprazole for 19 days. Significant differences were determined by factorial analysis of variance (*P < 0.05, **P < 0.01, ***P < 0.001 vs. either vehicle or gefitinib alone). Representative photographs show the external appearance of tumors. **g** PFS analysis between COX2-, MET-, and TOPK-positive and -negative patients with *EGFR*-activating mutations. *P *<* 0.05. Data are represented as the means ± SD of triplicate experiments. **h** Schematic diagram showing the mechanism of COX2/MET/TOPK signaling in gefitinib-resistant cells

As the inhibition of COX2 or TOPK could induce apoptosis of HCC827GR cells, we combined celecoxib (the COX2 inhibitor), pantoprazole (the TOPK inhibitor), and gefitinib in the following experiments. First, we assessed any potential cytotoxicity of gefitinib, celecoxib, and pantoprazole alone in HCC827GR cells (Fig. S2A). Then, combined effects were measured by MTT assay and analyzed using the Chou–Talalay method (Fig. S2B and C). The CI data showed that various concentrations of gefitinib, celecoxib, and pantoprazole induced different effects. The low-dose combination of gefitinib (1 μΜ), celecoxib (20 μΜ), and pantoprazole (100 μΜ) achieved a synergistic effect on proliferation inhibition in HCC827GR cells (Fig. S2C and Fig. 6a). As shown in Fig. 6b, this triple combination resulted in significantly higher inhibition of cell growth relative to any of the double combinations or single-drug treatments over 72 h. Subsequently, the triple treatment led to a significant induction in apoptosis as measured by PARP cleavage (Fig. 6c). Moreover, as expected, the triple combination was also shown to significantly inhibit the colony-formation ability of HCC827GR cells by using a soft agar assay (Fig. 6d). These results showed that the triple combination of drugs could suppress cell growth and colony formation accompanied by induction of apoptosis in vitro.

Based on the above results, we performed an animal study to test the effectiveness of the triple combination in preventing HCC827GR tumor growth. The results showed that the triple combination of gefitinib, celecoxib, and pantoprazole suppressed HCC827GR-mediated tumor growth (Fig. 6e, f). In addition, through retrospective analysis of 153 patients with *EGFR*-activating mutations, we identified that the PFS of gefitinib-resistant patients who were COX2, MET, and TOPK triple-positive was shorter than that of patients who were triple-negative (Fig. 6g). These results indicated that triple-positive patients with COX2, MET, and TOPK had a worse prognosis. Taken together, our study suggests that the triple combination of celecoxib, gefitinib, and pantoprazole can overcome gefitinib resistance.

## Discussion

It was reported that some factors, including hypoxia-induced factors, cytokines, HGF-dependent autocrine or paracrine loops, or proangiogenic factors, contributed to the transcriptional upregulation of MET^22,23^. From our data, overexpression of COX2 regulating MET expression at the transcriptional level via AP-1 induced gefitinib resistance. In addition, COX2 has served as a potential therapeutic target for lung cancer and selective COX2 inhibitors such as celecoxib were developed for use in lung cancer treatment and prevention^10^. Compared with those regulators of MET, the expression of COX2 could be a biomarker for gefitinib-resistant NSCLC with MET overexpression and COX2 could also be a target for treating gefitinib resistance.

In addition, COX2 is involved in various aspects of cancer primarily through PG synthesis and PGE_2_ might be the predominant PG in cancer^9^. However, in our study, TXA_2_ was the most represented PG in gefitinib-resistant cells compared with the other four major bioactive PGs and the COX2-TXA_2_ pathway-mediated MET transcription. It was reported that TXA_2_ or TXAS had been established as a tumor promoter in colon cancer or lung cancer^24^. Li et al.^25^ established that CRC progression is accompanied by elevated TXA_2_ levels and indicated that circulating TXA_2_ levels might have a potential prognostic or predictive value for the early detection of CRC. Hence, our findings suggested that TXA_2_ levels might have a potential predictive value in gefitinib-resistant NSCLC.

Drug resistance caused by MET overactivation is an urgent problem to be solved in the treatment of lung cancer. To date, some MET-targeted agents such as SU11274 and crizotinib have been developed and used in clinical experiments^26^. However, there have not been any useful drugs entering the market as first-line therapies due to negative or disappointing clinical trials^27^. One reason is that there are no appropriate clinical indicators for selecting patients with MET overactivation for treatment. MET protein expression was considered to have low predictive value, because the IHC assay lacked an established standard or consensus on optimized cutoff values and the total MET might not always reflect the activation of MET signaling^28^. In addition, only a few studies have investigated the expression of phosphorylated MET in lung cancer. Tretiakova et al.^29^ reported that the expression of two specific forms of p-MET (Y1003/Y1365) was associated with significantly worse overall survival. MET phosphorylation at Y1234/1235 and Y1349 could be detected in the NSCLC tumor samples, but there was no significant correlation between MET expression and p-MET (Y1234/1235 or Y1349) expression^30^. None of them could be used to select patients for targeting therapies. In our study, we showed that MET could be regulated by COX2 and could phosphorylate and activate TOPK at the Y74 site. Concurrently, the new COX2/MET/TOPK signaling axis promoted gefitinib resistance. Moreover, the PFS of COX2/MET/TOPK triple-positive patients with gefitinib resistance was shorter than that of triple-negative patients. In addition, the triple-positive patients with COX2, MET, and TOPK had a worse prognosis. Therefore, COX2 and TOPK expression can be associated with MET expression as predictive and prognostic markers for patients with gefitinib resistance.

The other reason that there is no useful treatment is that it is costly and time-consuming to explore new drugs for EGFR-TKI resistance. Our results strongly suggested that the combined inhibition of COX2, MET, and TOPK could overcome gefitinib resistance in HCC827GR cells, and that this strategy would be superior to targeting an individual pathway. Both celecoxib and pantoprazole are FDA-approved common drugs. Celecoxib, as a nonsteroidal anti-inflammatory drug, was reported to be used in the treatment of NSCLC due to the relationship between cancer and inflammation^31^. Moreover, pantoprazole was considered to increase the sensitivity of drug-resistant cancer cells such as lymphomas and gastric adenocarcinomas^32^. In our study, the triple combination approach with lower celecoxib, pantoprazole, and gefitinib concentrations effectively inhibited the tumorigenesis of gefitinib-resistant cells. Therefore, the new triple combination would provide a low-cost and practical strategy to help gefitinib-resistant patients.

In summary, our findings reveal a vital COX2/MET/TOPK signaling axis inducing gefitinib resistance in NSCLC and propose a low-cost and practical strategy to overcome this problem. In this regard, it might be useful to verify the effect of the triple combination of celecoxib, pantoprazole, and gefitinib through multicenter clinical trials, and to detect the TXA_2_ level in plasma, pleural effusions, or tissues of NSCLC patients and evaluate its clinical value.

## Supplementary information

Figure S1

Figure S2

supplementary materials and methods
