# Supplementary figures and images for: Targeting the COX2/MET/TOPK signaling axis induces apoptosis in gefitinib-resistant NSCLC cells
Source: Cell Death Dis. 2019 Oct 14;10(10):777. doi: 10.1038/s41419-019-2020-4 (PMC6791885; doi:10.1038/s41419-019-2020-4)

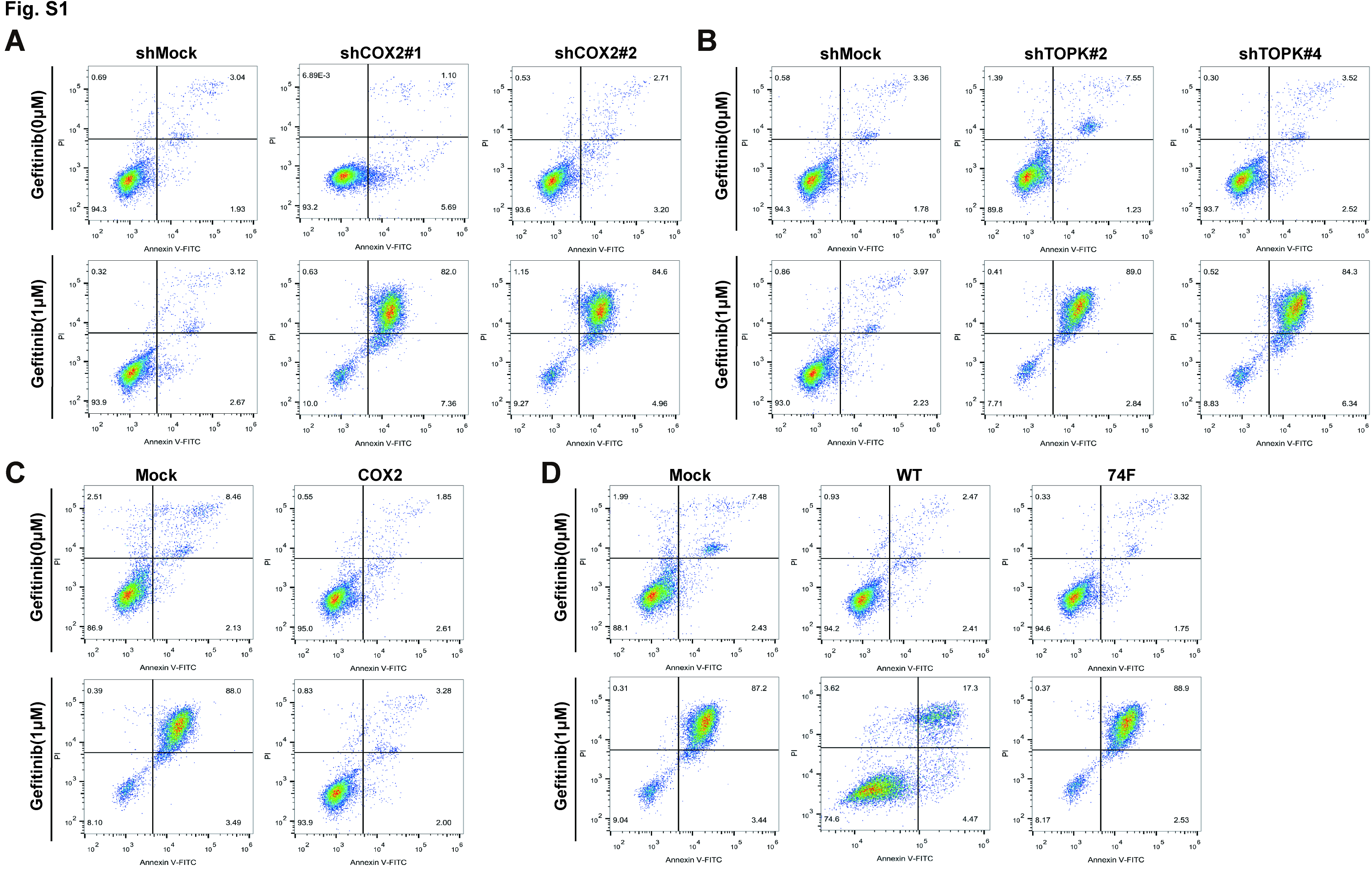

Supplement: Supplementary file 1 — Figure S1 [file 41419_2019_2020_MOESM1_ESM.tif]

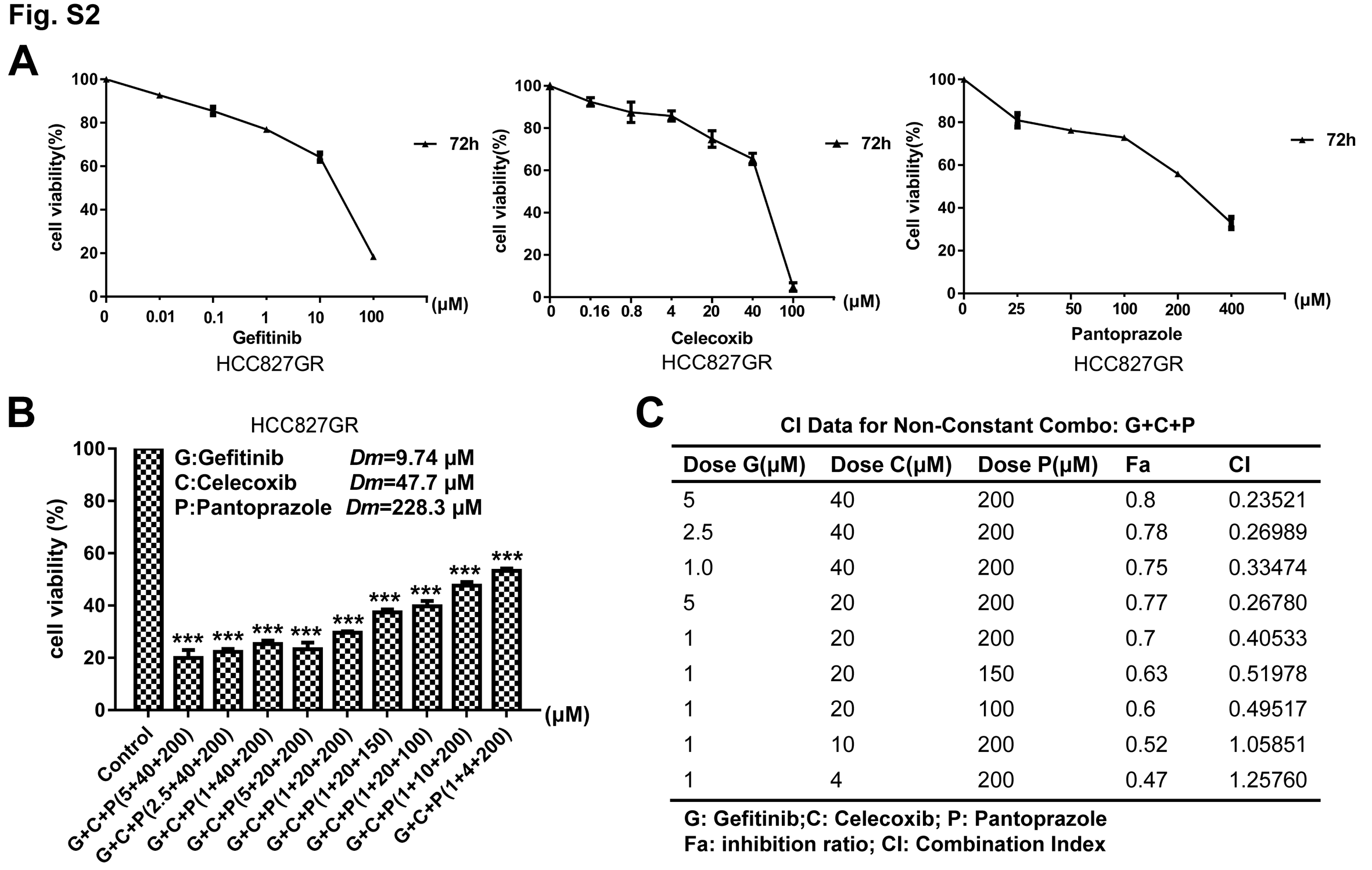

Supplement: Supplementary file 2 — Figure S2 [file 41419_2019_2020_MOESM2_ESM.tif]
