# Supplementary material for: Targeting the COX2/MET/TOPK signaling axis induces apoptosis in gefitinib-resistant NSCLC cells
Source: Cell Death Dis. 2019 Oct 14;10(10):777. doi: 10.1038/s41419-019-2020-4 (PMC6791885; doi:10.1038/s41419-019-2020-4)
Supplement: Supplementary file 3 — supplementary materials and methods [file 41419_2019_2020_MOESM3_ESM.doc]

**Supplementary Material and Methods**

**Construction of expression vectors**

Expression constructs, including Flag-COX2, His-MET were obtained from Sino Biological Inc (Beijing, BJ). HA-TOPK, pMD2.0G, and psPAX2 were purchased from Addgene (Cambridge, MA). Additionally, the lentivirus plasmids shCOX2 (#1, forward oligo, 5’ -CCGGGCTGAATTT

AACACCCTCTATCTCGAGATAGAGGGTGTTAAATTCAGCTTTTTTG-3’; #2, forward oligo, 5’- CCGGCCATTCTCCTTGAAAGGACTTCTCGAGAAGTCCTTTCAAGGAGAATG

GTTTTTTG -3’; #3, forward oligo, 5’- CCGGTGAGTACCGCAAACGCTTTATCTCGAGATA

AAGCGTTTGCGGTACTCATTTTTTG-3’; #4, forward oligo, 5’-CCGGCGTTGTGAATAACA

TTCCCTTCTCGAGAAGGGAATGTTATTCACAACGTTTTTTG-3’), shMET (#1, forward oligo, 5’- CCGGCCCGGATATCAGCGATCTTCTCTCGAGAGAAGATCGCTGATATCCGGG

TTTTTTG -3’; #2, forward oligo, 5’- CCGGGAAGTCCTCTTAACATCTATACTCGAGTATAG

ATGTTAAGAGGACTTCTTTTTTG-3’; #3, forward oligo, 5’-CCGGGCTGTGAGAATATAC

ACTTACCTCGAGGTAAGTGTATATTCTCACAGCTTTTTTG-3’; #4, forward oligo, 5’- CCGGCCTTCAGAAGGTTGCTGAGTA CTCGAGTACTCAGCAACCTTCTGAAGGTTTTT

G-3’), shTOPK (#2, forward oligo, 5’-CCGGGAAGTGTGGCTTGCGTAAATACTCGAGTATT

TACGCAAGCCACACTTCTTTTTG -3’; #4, forward oligo, 5’-CCGGGCCTTCATCATCCAA

ACATTGCTCGAGCAATGTTTGGATGATGAAGGCTTTTTG -3’) were obtained from Sangon Biotech, Inc. The pLKO.1-puro Non-Target shRNA Control Plasmid DNA (shMock) was purchased from Sigma-Aldrich. All constructs were confirmed by restriction enzyme mapping, DNA sequencing, and the Blast program.
